# Does the Amniotic Fluid of Mice Contain a Viable Microbiota?

**DOI:** 10.3389/fimmu.2022.820366

**Published:** 2022-02-28

**Authors:** Andrew D. Winters, Roberto Romero, Jonathan M. Greenberg, Jose Galaz, Zachary D. Shaffer, Valeria Garcia-Flores, David J. Kracht, Nardhy Gomez-Lopez, Kevin R. Theis

**Affiliations:** ^1^ Perinatology Research Branch, Division of Obstetrics and Maternal-Fetal Medicine, Division of Intramural Research, Eunice Kennedy Shriver National Institute of Child Health and Human Development, National Institutes of Health, U.S. Department of Health and Human Services, Detroit, MI, United States; ^2^ Perinatal Research Initiative in Maternal, Perinatal and Child Health, Wayne State University School of Medicine, Detroit, MI, United States; ^3^ Department of Biochemistry, Microbiology, and Immunology, Wayne State University School of Medicine, Detroit, MI, United States; ^4^ Department of Obstetrics and Gynecology, University of Michigan, Ann Arbor, MI, United States; ^5^ Department of Epidemiology and Biostatistics, Michigan State University, East Lansing, MI, United States; ^6^ Center for Molecular Medicine and Genetics, Wayne State University, Detroit, MI, United States; ^7^ Detroit Medical Center, Detroit, MI, United States; ^8^ Department of Obstetrics and Gynecology, Wayne State University School of Medicine, Detroit, MI, United States; ^9^ Department of Physiology, Wayne State University School of Medicine, Detroit, MI, United States; ^10^ MD/PhD Combined Degree Program, Wayne State University School of Medicine, Detroit, MI, United States

**Keywords:** amniotic fluid, microbiome, microbiota, *in utero* colonization, sterile womb hypothesis, mouse model, low microbial biomass study

## Abstract

The existence of an amniotic fluid microbiota (i.e., a viable microbial community) in mammals is controversial. Its existence would require a fundamental reconsideration of fetal *in utero* exposure to and colonization by microorganisms and the role of intra-amniotic microorganisms in fetal immune development as well as in pregnancy outcomes. In this study, we determined whether the amniotic fluid of mice harbors a microbiota in late gestation. The profiles of the amniotic fluids of pups located proximally or distally to the cervix were characterized through quantitative real-time PCR, 16S rRNA gene sequencing, and culture (N = 21 dams). These profiles were compared to those of technical controls for bacterial and DNA contamination. The load of 16S rRNA genes in the amniotic fluid exceeded that in controls. Additionally, the 16S rRNA gene profiles of the amniotic fluid differed from those of controls, with *Corynebacterium tuberculostearicum* being differentially more abundant in amniotic fluid profiles; however, this bacterium was not cultured from amniotic fluid. Of the 42 attempted bacterial cultures of amniotic fluids, only one yielded bacterial growth – *Lactobacillus murinus*. The 16S rRNA gene of this common murine-associated bacterium was not detected in any amniotic fluid sample, suggesting it did not originate from the amniotic fluid. No differences in the 16S rRNA gene load, 16S rRNA gene profile, or bacterial culture were observed between the amniotic fluids located Proximally and distally to the cervix. Collectively, these data indicate that, although there is a modest DNA signal of bacteria in murine amniotic fluid, there is no evidence that this signal represents a viable microbiota. While this means that amniotic fluid is not a source of microorganisms for *in utero* colonization in mice, it may nevertheless contribute to fetal exposure to microbial components. The developmental consequences of this observation warrant further investigation.

## Introduction

The mammalian amniotic cavity is filled with a protective liquid (i.e., amniotic fluid) that surrounds the fetus throughout gestation. Indeed, the amniotic fluid is essential for fetal development and maturation (1, 2). As such, the amniotic fluid is enriched with nutrients and growth factors (1, 3–5) and contains soluble [e.g. cytokines (6–27), anti-microbial molecules, etc. (28–33)] and cellular [e.g. innate and adaptive immune cells (34–40)] components that serve as an immunological barrier against invading pathogens. In clinical medicine, amniotic fluid is utilized as a diagnostic tool for assessing intra-amniotic inflammation and/or infection (41–59), a condition that is strongly associated with obstetrical disease, the most detrimental of which is preterm birth (60). Therefore, the presence of microorganisms in the amniotic fluid is associated with adverse maternal and neonatal outcomes (61–68), and the traditional view in obstetrics has been the “sterile womb hypothesis”, which posits that the fetal environment is sterile and that the neonate first acquires a microbiota during the birthing process (69). However, recent investigations have posited that the placenta (70–82), the amniotic fluid (75, 83–85), and the developing fetus (86–88) harbor resident microbiotas, and that the amniotic fluid microbiota functions as a primary source of microorganisms for initial colonization of the offspring *in utero* (75, 83, 85, 89, 90). These juxtaposed views have sparked much debate (69, 84, 91–95).

Investigations of human amniotic fluid in normal pregnancy have yielded contradictory results. Multiple studies using DNA sequencing techniques (75, 84, 85, 96–98) and/or quantitative real-time PCR (83, 97) have identified a molecular signal indicating the presence of an amniotic fluid microbiota; however, only one of these studies has demonstrated viable microorganisms from amniotic fluid through culture (75) (**Supplementary Table 1**). To date, no study has used cultivation, qPCR, and DNA sequencing concurrently to confirm the existence of a microbiota in human amniotic fluid during normal pregnancy. The concurrent use of multiple microbiological techniques in such investigations is important because a molecular signal of microorganisms is not necessarily equivalent to a true and viable microbiota (69, 83, 99–101). For instance, the molecular signal may simply reflect circulating microbial DNA fragments (102). Furthermore, if there is an amniotic fluid microbiota, it has a very low microbial biomass and, therefore, reliance on molecular techniques such as DNA sequencing to characterize the presumed microbiota is susceptible to influences of background DNA contamination from laboratory environments, DNA extraction kits, PCR reagents, etc. (103). Yet, very few of the prior investigations that used DNA sequencing techniques to conclude the existence of a human amniotic fluid microbiota incorporated technical controls for background DNA contamination into their analyses (84, 97, 98, 104, 105) (**Supplementary Table 1**). Hence, there remains uncertainty as to whether the human amniotic fluid and the intra-uterine environment, in general, harbor a microbiota.

The existence of an amniotic fluid microbiota would require a fundamental reconsideration of the role of intra-amniotic microorganisms in fetal development and pregnancy outcomes. Such reconsideration would require the implementation of animal models to perform mechanistic experimentation of host immune-microbe interactions. Yet, there have been only a limited number of studies investigating the presence of an amniotic fluid microbiota in animal models, specifically cattle, horses, sheep, goats, and rats (**Supplementary Table 2**). Although each of these studies used DNA sequencing techniques, very few included qPCR, technical controls for background DNA contamination, or culture. Therefore, the objective of the current study was to determine whether the amniotic fluid of mice, the most widely utilized system for studying host immune-microbe interactions (106), harbors a viable microbiota by using technical controls, qPCR, 16S rRNA gene sequencing, and bacterial culture.

## Materials and Methods

### Study Subjects

C57BL/6 mice were obtained from The Jackson Laboratory (Bar Harbor, ME, USA) and bred at the C.S. Mott Center for Human Growth and Development at Wayne State University, Detroit, MI, USA in the specific-pathogen-free (SPF) animal care facility. Mice were housed under a 12 h:12 h light/dark cycle and had access to food (PicoLab laboratory rodent diet 5L0D; LabDiet, St. Louis, MO, USA), and water *ad libitum*. Females (8-12 weeks old) were mated with males of demonstrated fertility. Daily examination was performed to assess the appearance of a vaginal plug, which indicated 0.5 days *post coitum* (dpc). Dams were then housed separately from the males and their weights were checked daily. An increase in weight of ≥ 2 g by 12.5 dpc confirmed pregnancy. All procedures were approved by the Institutional Animal Care and Use Committee (IACUC) (Protocol No. 18-03-0584) of Wayne State University.

### Sample Collection and Storage

**Figure 1:**
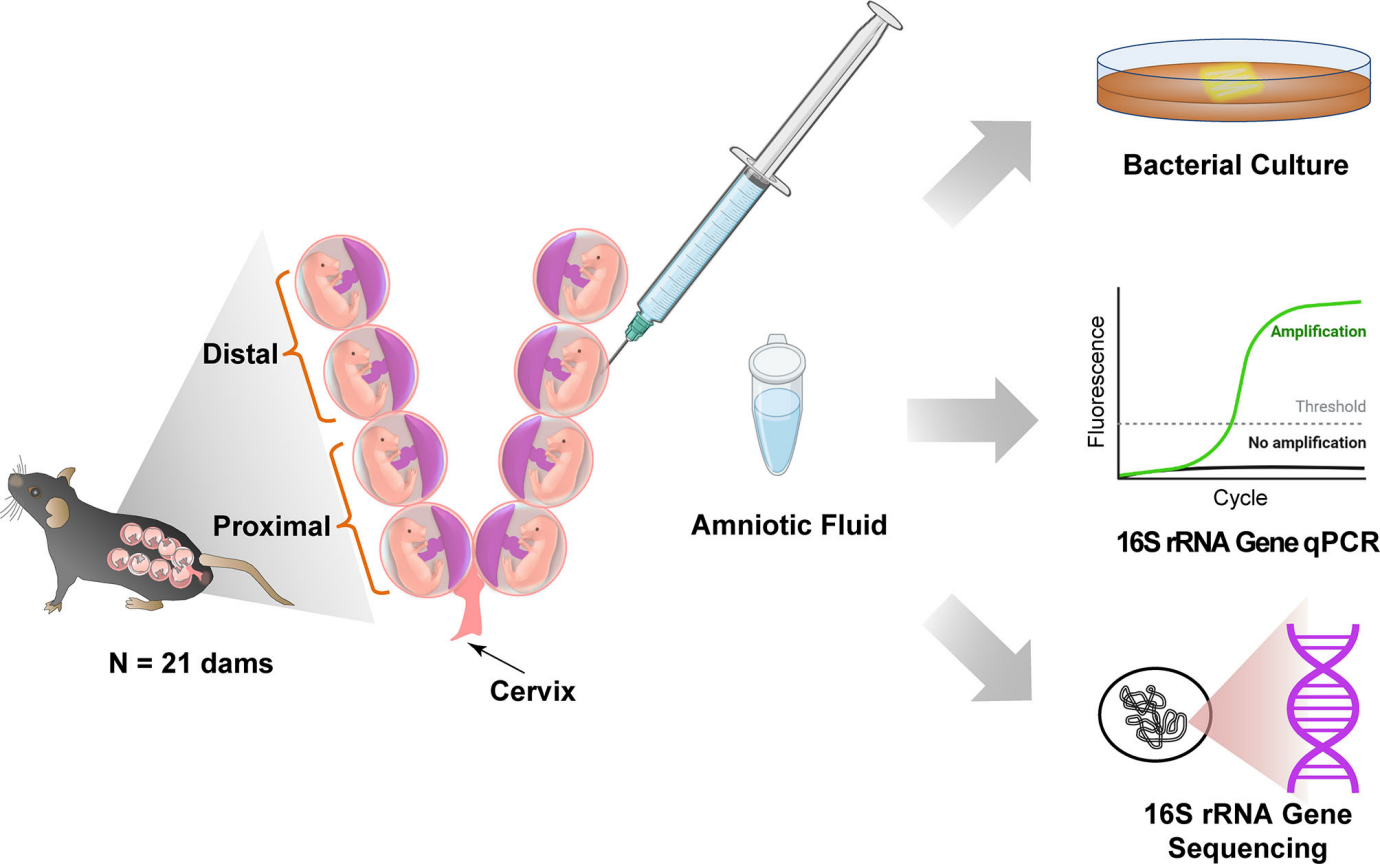
Study design to test for the presence of bacteria in murine amniotic fluid. Created with BioRender.com.

Twenty-one pregnant mice were included in this study (**Figure 1**). Pregnant mice were euthanized during the second half of pregnancy (13.5-18.5 dpc). The abdomen was shaved, and 70% ethanol was applied. Dams were placed on a sterile surgical platform within a biological safety cabinet. Study personnel wore sterile sleeves, masks, and powder-free sterile gloves during sample collection, and sterile disposable scissors and forceps were utilized. Iodine was applied to the abdomen with a sterile cotton swab, and after the iodine dried, a midline skin incision was performed along the full length of the abdomen. The peritoneum was longitudinally opened, using a new set of scissors and forceps, and the uterine horns were separated from the cervix and placed within a sterile petri dish. A sterile syringe with a 26G needle was utilized to obtain amniotic fluid from amniotic sacs proximal to the cervix and from amniotic sacs distal from the cervix. Due to the small volume of amniotic fluid often obtained from each amniotic sac (< 40 µl), amniotic fluid was obtained from two adjoining amniotic sacs and pooled. The amniotic fluid was aliquoted into two sterile tubes and transported immediately to the microbiology lab for bacterial culture and molecular analyses, respectively. The tube with the amniotic fluid for molecular analyses was stored at −80°C.

### Culture of Amniotic Fluid Samples

For all mice, proximal and distal amniotic fluid samples (~40 µL each) were cultured in 200 µL of Brain-Heart-Infusion (BHI) broth supplemented with 5 mg/L of hemin and 2 µg/L of vitamin K1 under oxic and anoxic conditions for 48 hours. For the first eight mice in the study, 40 µL of the BHI culture were then plated on supplemented BHI agar plates and cultured under the respective atmospheric condition for an additional 48 hours, and resultant bacterial isolates were taxonomically characterized. For the last 13 mice in the study, 40 µL of the BHI culture were subsequently plated on supplemented BHI agar plates and cultured under the respective atmospheric condition if turbidity of the broth culture was observed after 48 hours of incubation. Any potential growth of bacteria in BHI broth cultures of proximal and distal amniotic fluid samples was then assessed through qPCR and 16S rRNA gene sequencing. As each amniotic fluid sample was cultured under oxic and anoxic conditions, 125 µL each from the oxic and anoxic broth cultures were pooled and stored at −80°C. The 16S rRNA gene loads and profiles of these amniotic fluid broth cultures were compared to those of six uninoculated BHI broth negative controls using qPCR and 16S rRNA gene sequencing.

Secondarily, after finding that DNA from the 16S rRNA gene of *Corynebacterium tuberculostearicum*, in particular, was more relatively abundant in proximal and distal amniotic fluid samples than in background technical controls (see *Results* section, **Figure 3**), we validated our culture protocol for the recovery and growth of *C. tuberculostearicum*. The *C. tuberculostearicum* type strain ATCC 35692 was ordered, and the freeze-dried pellet was recovered in approximately 5mL of BHI broth. After 48 hours, growth from the primary inoculum was separated into 500 µl aliquots with glycerol at a concentration of 17.5% and frozen at -80°C for subsequent culture validation. Initially, 40 µl of the frozen ATCC stock were inoculated into 400 µl of BHI broth and then five 10-fold serial dilutions were performed. Tubes were incubated for 48 hours under aerobic conditions at 37°C and 40 µl of the 48-hour enrichment broth were plated onto BHI agar plates for an additional 48 hours of incubation. Colony counts were recorded and CFU (colony forming units) per mL and CFU limits of detection were calculated. All cultures were performed in duplicate and were CFU counts were averaged between the duplicate plates. Colony counts of the stock cultures indicated the original stock contained approximately 976,250 CFU/mL. Following 48 hours of broth enrichment, plated cultures were countable for the 10^-3^ (630 CFUs) and 10^-4^ dilutions (11 CFUs); no colonies were observed for the 10^-5^ dilution. After backcalculating the dilution and original inoculum volume, approximately four CFUs were inoculated into the 10^-4^ broth dilution tube, indicating that the limit of detection for *C. tuberculostearicum* was four CFUs.

### DNA Extraction

Genomic DNA was extracted within a biological safety cabinet from amniotic fluid and BHI broth samples, as well as positive [i.e., human clean catch urine (N=3)] and negative [i.e., blank DNA extraction kit (N=14), sterile BHI broth (N=6)] controls using the DNeasy PowerLyzer Powersoil kit (Qiagen, Germantown, MD, USA), with minor modifications to the manufacturer’s protocols as previously described (107, 108). Specifically, following UV treatment, 400 µL of Powerbead solution, 200 µL of phenol:chloroform:isoamyl alcohol (pH 7-8), and 60 µL of preheated solution C1 were added to the provided bead tubes. Next, 250 µL of an amniotic fluid or of a BHI sample were added to the tubes. When less than 250 µL of amniotic fluid were available (9/41 samples, 21%) a minimum of 100 µL was added. Tubes were briefly vortexed, and cells were mechanically lysed in a bead beater for two rounds of 30 sec each. Following 1 minute of centrifugation, supernatant was transferred to new tubes and 1 µL of PureLink™ RNase A (20mg/mL, Invitrogen Carlsbad, CA, USA), 100 µL of solution C2, and 100 µL of solution C3 were added. Tubes were then incubated at 4°C for 5 min. After a 1 min centrifugation, lysates were transferred to new tubes containing 650 µL of C4 solution and 650 µL of 100% ethanol. Lysates were then loaded onto filter columns 635 µL at a time, centrifuged for 1 min, and the flowthrough discarded. This wash process was repeated three times to ensure all lysate passed through the filter columns. Following the wash steps, 500 µL of solution C5 was added to the filter columns and centrifuged for 1 min. After discarding the flowthrough, the tubes were centrifuged for 2 min to dry the filter columns. The spin columns were transferred to clean 2.0 mL collection tubes and 60 µL of pre-heated solution C6 were added directly to the center of the spin columns. Following a 5 min room temperature incubation, DNA was eluted by centrifuging for 1 min. Purified DNA was then transferred to new 2.0 mL collection tubes and stored at −20°C.

### 16s rRNA Gene Quantitative Real-Time PCR

To measure total 16S rRNA gene abundance within samples, amplification of the V1-V2 region of the 16S rRNA gene was performed according to the protocol of Dickson et al. (109), with minor modifications as previously described (107, 108). The modifications consisted of using a degenerative forward primer (27f-CM: 5’-AGA GTT TGA TCM TGG CTC AG-3’) and a degenerate probe with locked nucleic acids (+) (BSR65/17: [5’-56FAM-TAA +YA+C ATG +CA+A GT+C GA-BHQ1-3’]). Each 20 µL reaction was performed with 0.6 µM of 27f-CM primer, 0.6 µM of 357R primer (5’-CTG CTG CCT YCC GTA G-3’), 0.25 μM of BSR65/17 probe, 10.0 µL of 2X TaqMan Environmental Master Mix 2.0 (Invitrogen), and 3.0 µL of purified DNA or nuclease-free water. The following conditions were used to perform the total bacterial DNA qPCR: 95° C for 10 min, and then 40 cycles of 94°C for 30 sec, 50°C for 30 sec, and 72°C for 30 sec. Each reaction was performed in triplicate using an ABI 7500 thermocycler (Applied Biosystems, Foster City, CA, USA). After normalization to the ROX passive reference dye, the 7500 Software version 2.3 (Applied Biosystems, Foster City, CA, USA) was used to analyze the raw amplification data with the default threshold and baseline settings. Calculation of the cycle of quantification (Cq) values for the samples was based upon the mean number of cycles necessary for the exponential increase of normalized fluorescence.

### 16s rRNA Gene Sequencing

The V4 region of the 16S rRNA gene was amplified and sequenced *via* the dual indexing strategy developed by Kozich et al. (110). The forward and reverse primers used were 515F: 5’-GTGCCAGCMGCCGCGGTAA-3’ and 806R: 5’-GGACTACHVGGGTWTCTAAT-3’, respectively. Duplicate 20 µL PCR reactions were performed containing 0.75 µM of each primer, 3.0 µL DNA template, 10.0 µL of DreamTaq High Sensitivity Master Mix (Thermo Scientific, Waltham, MA, USA), and 5 µL of DNase-free water. Reaction conditions were as follows: 95° for 3 min, followed by 38 cycles of 95°C for 45 sec, 50°C for 60 sec, and 72°C for 90 sec, followed by an additional elongation at 72°C for 10 min. The duplicate PCR reactions were then pooled, and DNA was quantified with a Qubit 3.0 fluorometer and the Qubit dsDNA assay kit (Life Technologies, Carlsbad, CA, USA) following the manufacturer’s protocol. Samples were pooled in equimolar concentrations and purified by using the Cytiva Sera-Mag Select DNA Size Selection and PCR Clean-Up Kit (Global Life Sciences, Little Chalfont, Buckinghamshire, UK), according to the manufacturer’s instructions. Illumina MiSeq sequencing was performed at the Michigan State University Research Technology Support Facility Genomics Core. Specifically, sequencing was performed in a 2x250bp paired end format using a 500 cycle v2 reagent cartridge.

Raw sequence reads were processed by using DADA2 (v 1.12) (111). An analysis of 16S rRNA gene amplicon sequence variants (ASVs), defined by 100% sequence similarity, was performed using DADA2 in R (v 3.5.1) (https://www.R-project.org) and the online MiSeq protocol (https://benjjneb.github.io/dada2/tutorial.html) with minor modifications. These modifications included allowing truncation lengths of 250 bp and 150 bp and a maximum number of expected errors of 2 bp and 7 bp for forward and reverse reads, respectively. To allow for increased power to detect rare variants, sample inference allowed for the pooling of samples. Additionally, samples in the resulting sequence table were pooled prior to removal of chimeric sequences. Sequences were then classified by using the silva_nr_v132_train_set database with a minimum bootstrap value of 80%, and sequences derived from Archaea, chloroplast, or Eukaryota were removed.

The R package *decontam* version 1.6.0 (112) was used to identify ASVs that were likely potential background DNA contaminants based on their distribution among biological samples (amniotic fluid and BHI cultures) and negative controls (blank DNA extractions and stock BHI broth) using the “IsNotContaminant” method. Identification of contaminant ASVs was assessed for amniotic fluid and BHI cultures independently. An ASV was determined to be a contaminant, and was removed from the dataset, if it had a *decontam* P score ≥ 0.7 and was present in at least 20% of the negative controls with an overall average relative abundance of at least 1.0%.

### Statistical Analysis

Prior to statistical analyses, the bacterial profiles of proximal and distal amniotic fluid samples and blank DNA extraction controls were rarefied to 1,366 sequence reads (set.seed = 1) using phyloseq (113). The bacterial profiles of proximal and distal BHI culture samples and stock BHI broth samples were rarefied to 21,227 sequence reads. Variation in the bacterial profiles was visualized through Principal Coordinates Analyses (PCoA) using the R package vegan version 2.5-6 (114). Alpha diversity values and 16S rRNA gene signal (qPCR Cq) values across sample groups were compared by using the “wilcox.test” function in R version 3.6.0. Beta diversity of amniotic fluid bacterial profiles was characterized using the Bray-Curtis dissimilarity index. Bacterial community structure of amniotic fluid and BHI culture samples was compared by using PERMANOVA (115) with the “adonis” function in the R package vegan version 2.5-6 (114). Assessment of differentially abundant taxa across sample groups was performed by using Linear Discriminant Analysis Effect Size, or LEfSe (116), with default parameters. Analysis of the phylogenetic relationships of selected ASVs and other bacteria was performed using the Neighbor-Joining method (117) in MEGA 6 software (118) with the Maximum Composite Likelihood method and bootstrapping of 1,000 replicates, allowing for transitions and transversions.

## Results

### Murine Amniotic Fluid Contains Bacterial 16s rRNA Gene Copies

**Figure 2:**
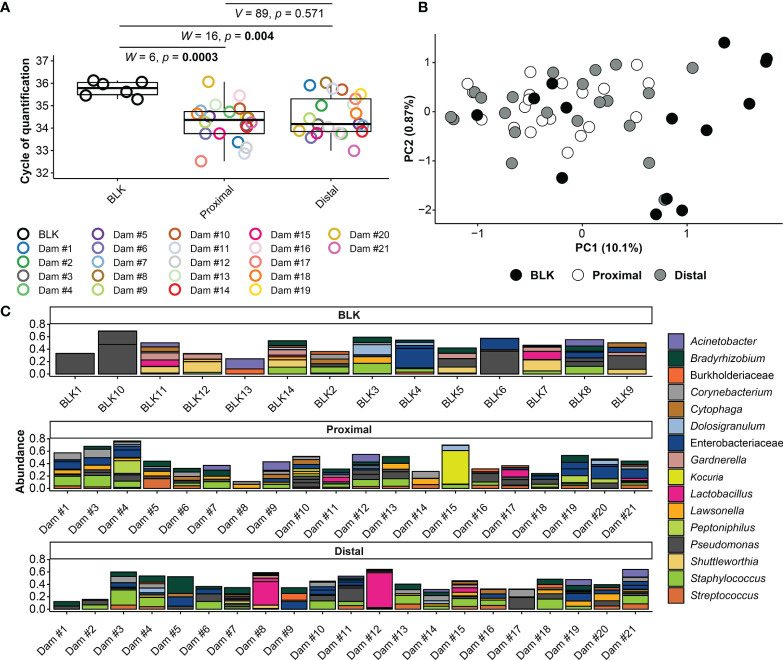
16S rRNA gene qPCR and sequencing results for amniotic fluid and blank control samples. **(A)** Cycle of Quantification (Cq) values from qPCR of proximal and distal amniotic fluid and blank control (BLK) samples. **(B)** Principal coordinate analysis (PCoA) illustrating variation in 16S rRNA gene profiles among proximal and distal amniotic fluid and blank control samples. Similarities in the 16S rRNA gene profiles were characterized using the Bray-Curtis similarity index. **(C)** Taxonomic classifications of the 20 amplicon sequence variants (ASVs) with highest relative abundance across all proximal and distal amniotic fluid and blank control samples. Bars of identical color within the same sample indicate multiple ASVs with the same bacterial taxonomic classification. The DNA extract of the proximal amniotic fluid sample from Dam #2 did not yield a 16S rRNA gene sequence library.

Amniotic fluid was collected from amniotic sacs located proximally and distally to the cervix under aseptic conditions from 13.5 – 18.5 dpc (**Figure 1**). First, we evaluated the absolute abundance of bacterial 16S rRNA gene copies in amniotic fluid using qPCR. There was a significantly higher 16S rRNA gene signal in proximal (*W* = 6, *p* = 0.0003) and distal (*W* = 16, *p* = 0.004) amniotic fluid samples than in blank extraction controls. However, the 16S rRNA gene signal did not differ between paired proximal and distal samples (*V* = 89, *p* = 0.571) (**Figure 2A**). These results indicate that the murine amniotic fluid contains 16S rRNA gene copies, and that their concentrations do not depend on proximity to the cervix.

### 16s rRNA Gene Profiles Differ Between Murine Amniotic Fluid and Controls

**Figure 3:**
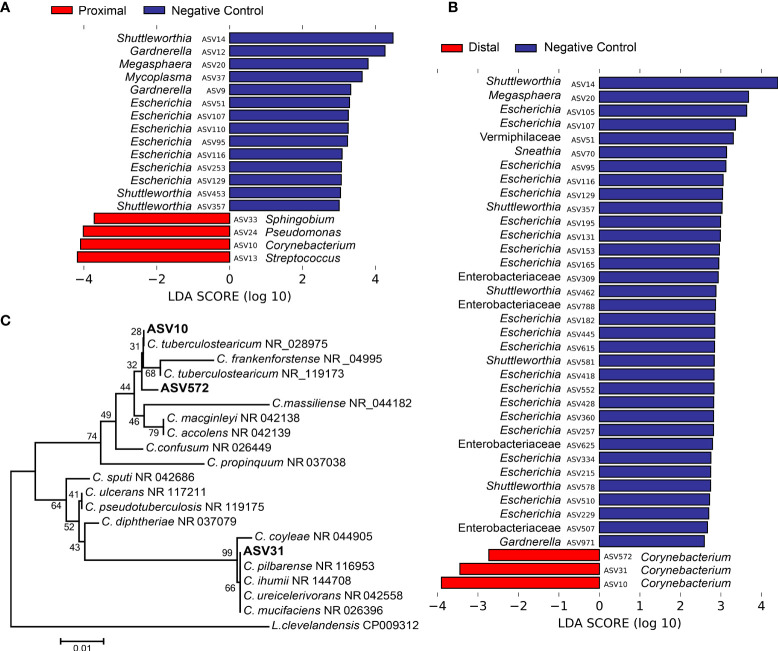
Differentially abundant amplicon sequence variants (ASVs) in proximal and distal amniotic fluid and blank control samples. **(A)** proximal and **(B)** distal amniotic fluid samples compared to blank DNA extraction controls as determined by Linear discriminant analysis effect size analyses. **(C)** Dendrogram of the three differentially abundant *Corynebacterium* ASVs in amniotic fluid samples and partial 16S rRNA gene sequences of closely related bacterial type strains. Numbers at the nodes are maximum-likelihood bootstrap values. Scale bar indicates the number of nucleotide substitutions per site.

Next, the 16S rRNA gene profiles of the amniotic fluid samples were characterized by using nucleotide sequencing and the generation of amplicon sequence variants (ASVs). Prior to removing potential background DNA contaminants, the 16S rRNA gene profiles of both the proximal and distal amniotic fluid samples differed from that of negative controls (PERMANOVA *F* = 2.343, *R* ^2^ = 0.068, *p* = 0.0001 and *F* = 1.806, *R* ^2^ = 0.052, *p* = 0.008, respectively) (**Figure 2B**). The most prominent ASVs in the proximal and distal amniotic fluid samples and technical controls were *Staphylococcus*, *Pseudomonas*, and *Enterobacteriaceae* (**Figure 2C**). There were differentially abundant taxa between the amniotic fluid samples and negative controls (**Figures 3A, B**). Most notably, multiple ASVs classified as *Corynebacterium* were more abundant in proximal (ASV 10) and distal (ASVs 10, 31 and 572) amniotic fluid samples than in controls (**Figures 3A, B**). These corynebacteria were most closely related to *C. tuberculostearicum*, *C*. *mucifaciens*, *C*. *ureicelerivorans*, *C*. *ihumii*, and C. *pilbarense* (**Figure 3C**). Additional taxa that were differentially abundant in proximal amniotic fluid samples compared to controls were *Streptococcus* (ASV 13), *Pseudomonas* (ASV 24), and *Sphingobium* (ASV 33) (**Figure 3A**).

**Figure 4:**
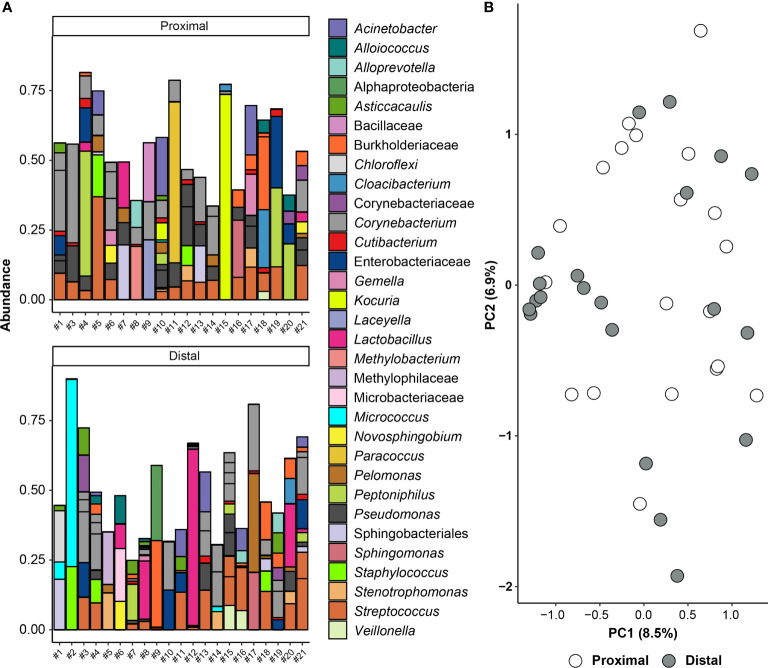
Amniotic fluid sequencing results after the removal of likely contaminating sequences. **(A)** Bar graph showing the taxonomy of the 45 amplicon sequence variants (ASVs) with highest relative abundance across all proximal and distal amniotic samples. Bars of identical color within the same sample indicate multiple ASVs with the same bacterial taxonomic classification. The DNA extract of the proximal amniotic fluid sample from Dam #2 did not yield a 16S rRNA gene sequence library. **(B)** Principal coordinate analysis (PCoA) illustrating variation in 16S rRNA gene profiles among proximal and distal amniotic fluid samples. The 16S rRNA gene profiles were characterized using the Bray-Curtis similarity index.

To address whether the 16S rRNA gene signals in amniotic fluid may represent potential background DNA contamination, the R package *decontam* was used to identify and remove likely contaminants. After contaminants were removed from the dataset, the ASVs with the highest mean relative abundance in both proximal and distal amniotic fluid samples were *Corynebacterium* and *Streptococcus* (**Figure 4A**). This contrasts with the profile structure before contaminant removal (**Supplemental Figure 1**). The 16S rRNA gene profiles of paired proximal and distal amniotic fluid samples did not differ in richness (Chao1 richness) (*V* = 58, *p* = 0.083) or in evenness (Shannon-Wiener diversity) (*V* = 76, *p* = 0.294). The structure of these profiles did not differ either by mouse dam ID (PERMANOVA *F* = 0.992, *R* ^2^ = 0.495, *p* = 0.551) or proximity to the cervix (*F* = 1.215, *R* ^2^ = 0.030, *p* = 0.089) (**Figure 4B**). Collectively, these results indicate that, if there is a murine amniotic fluid microbiota, it is largely comprised of *Corynebacterium* and *Streptococcus*, both of which are readily grown on brain heart infusion medium (119, 120), which was the medium used for the culture component of this study (see *Materials and Methods* section *Culture of Amniotic Fluid Samples*).

### Murine Amniotic Fluid Does Not Contain a Viable Microbiota

**Figure 5:**
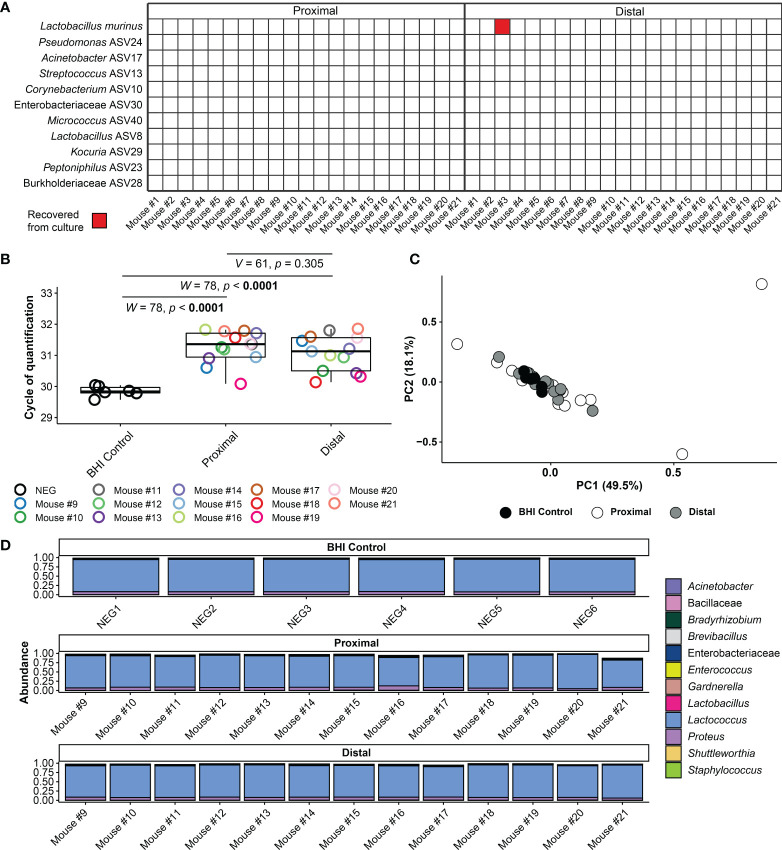
Amniotic fluid culture and blank control 16S rRNA gene qPCR and sequencing results. **(A)** Bacterial cultivation results for proximal and distal amniotic fluid samples. **(B)** Cycle of quantification values from qPCR on amniotic fluid culture samples and BHI culture medium controls. **(C)** Principal coordinate analysis (PCoA) of bacterial relative abundance data from amniotic fluid samples and BHI culture medium controls. **(D)** Relative abundance of bacteria in the 16S rRNA gene profiles of amniotic fluid samples and BHI culture medium controls. Bars of identical color within the same sample indicate multiple amplicon sequence variants with the same bacterial taxonomic classification.

Forty-two amniotic fluid samples were cultured for bacteria, and only one amniotic fluid sample (Dam #3 distal) yielded bacterial growth (**Figure 5A**). For this sample, multiple colonies of a single bacterial morphotype (Gram positive rod) were ultimately recovered under oxic and anoxic conditions. The partial 16S rRNA genes (703 bp) of these isolates were at least 99.7% identical to *Lactobacillus murinus* NBRC 14221 (NR_112689). The distal amniotic fluid sample from Dam #3 did not have 16S rRNA gene concentrations outside the range of other amniotic fluid samples in the study (**Figure 2A**), which would be expected if it was truly and uniquely populated with *L. murinus*.

Secondarily, for 13/21 dams (i.e., the last 13 dams sampled), we further characterized the 16S rRNA gene concentration and profile of the amniotic fluid-inoculated BHI broths and compared these data to those of stock control broth. Overall, the 16S rRNA gene signal of inoculated broths did not exceed that of stock control broth (**Figure 5B**). Additionally, the 16S rRNA gene profile of both the proximal and distal amniotic fluid cultures did not differ from those of the stock BHI control broth (PERMANOVA *F* = 0.702, *R* ^2^ = 0.04, *p* = 0.602 and *F* = 0.918, *R* ^2^ = 0.051, *p* = 0.461, respectively) (**Figures 5C, D**). Like the data for 16S rRNA gene concentration (**Figure 5B**), the 16S rRNA gene profile did not differ between paired proximal and distal amniotic fluid inoculated broths (**Figures 5C, D**). After removal of contaminants from the dataset using *decontam*, only one-half of the paired amniotic fluid culture samples (N = 7) had at least 500 sequence reads remaining (i.e., the majority of sequence data from amniotic fluid inoculated broths was identified as likely DNA contamination). The structures of the proximal and distal culture 16S rRNA gene profiles did not vary by mouse dam ID (PERMANOVA *F* = 0.815, *R* ^2^ = 0.409 *p* = 0.807) or differ based on proximity to the cervix (*F* = 1.057, *R* ^2^ = 0.089, *p* = 0.317).

Taken together, using culture and molecular interrogations of culture broths, these data provide no evidence of bacterial growth in proximal or distal amniotic fluids.

## Discussion

In the current study, we utilized qPCR, 16S rRNA gene sequencing, and bacterial culture to investigate the presence and consistency of bacterial signals in murine amniotic fluids. Molecular techniques indicated the presence of a 16S rRNA gene signal in the amniotic fluids, yet this signal was not verified through culture as coming from a viable microbiota.

### Prior Reports of an Amniotic Fluid Microbiota in Normal Human Pregnancy

Investigations that used qPCR, 16S rRNA gene sequencing, or cultivation to determine the presence of a human amniotic fluid microbiota in normal pregnancy have yielded inconsistent findings (75, 83–85, 96–98, 104, 105, 121). This is likely due in part to insufficient methods such as a lack of multiple complementary techniques for bacterial detection and characterization and/or a lack of appropriate technical controls. Notably, of these studies, only one reported the isolation of bacteria (Propionibacterium [Cutibacterium] and Staphylococcus) from human amniotic fluid of women who delivered a term neonate (75). These bacteria were also identified in the 16S rRNA gene profiles of amniotic fluid; however, they are typical inhabitants of the human skin and may therefore represent skin contaminants introduced during cesarean delivery (122).

Overall, of the studies which performed 16S rRNA gene sequencing to investigate the existence of a human amniotic fluid microbiota in normal pregnancies (75, 84, 85, 96–98, 104, 105, 121), only five included technical controls for background DNA contamination (84, 97, 98, 104, 105). Three concluded the existence of an amniotic fluid microbiota, although these studies did not include a culture component (84, 97, 98). The first study (84) reported that 83.7% (36/43) of amniotic fluid samples had a 16S rRNA gene signal, with varying degrees of *Propionibacterium* (*Cutibacterium*) *acnes*, *Staphylococcus epidermidis*, *Ralstonia*, *Streptococcus anginosus*, and *Peptoniphilus* dominance. The second study (97) reported that 19.9% (238/1,198) of amniotic fluid samples yielded a 16S rRNA gene signal; they were dominated by Saccharibacteria, *Acidovorax*, *Tepidimonas*, *Pelomonas*, and *Streptococcus oligofermentans*. In the third study (98), only 13.8% (4/29) of amniotic fluid samples had a detectable 16S rRNA gene signal, with *Actinomyces*, *Cutibacterium*, *Staphylococcus*, and *Streptococcus* being most relatively abundant. Thus, the most reported bacterial taxa detected in human amniotic fluid investigations were *Staphylococcus* and *Cutibacterium*, two typical skin bacteria (122). These results illustrate the need for more comprehensive investigations that implement multiple complementary modes of microbiologic inquiry as well as the need for robust technical controls.

### Existence of an Amniotic Fluid Microbiota in Animal Models

In cattle, three investigations utilized 16S rRNA gene sequencing to explore the presence of an amniotic fluid microbiota (123–125) (**Supplementary Table 2**). Two concluded the existence of an amniotic fluid microbiota, using this approach (123, 124); however, one study, which also included qPCR, and culture, concluded that the bacterial signals in the amniotic fluid did not exceed those in controls (125). In two investigations of horses and goats, a microbiota was identified in the amniotic fluid using 16S rRNA gene sequencing (126, 127). However, in a study of sheep, the amniotic fluid was determined to be sterile using qPCR, and 16S rRNA gene sequencing (128). Clearly, in bovids and equids, the existence of an amniotic fluid microbiota remains uncertain.

In the only study to date of rodents (89), 16S rRNA gene sequencing was used to demonstrate that amniotic fluid microbiota profiles were pup- and dam-specific in a rat model, yet they were not different from those of the placenta or fetal intestine. The primary bacteria detected were identified as Lachnospiraceae, Ruminococcaceae, Bacteroidaceae, Veillonellaceae, Rikenellaceae, and Propionibacteriaceae (89). However, this study did not include qPCR, or culture components, so it is not clear whether these 16S rRNA gene signals represent a viable amniotic fluid microbiota.

### Our Findings in the Context of Prior Studies

The *in utero* colonization hypothesis posits that developing fetuses are colonized by microorganisms prior to labor and delivery. Two recent studies provided evidence for *in utero* colonization in humans (87, 88). In the first study (87), fluorescence *in situ* hybridization and scanning electron microscopy revealed sparse patches of bacteria-like structures in the intestinal meconium of second trimester fetuses from terminated pregnancies. 16S rRNA gene sequencing identified Micrococcaceae and *Lactobacillus* as being enriched in the meconium, and *Micrococcus* was ultimately cultured from it. In the second study (88), bacteria-like structures were again visualized in intestinal samples from second trimester fetuses from terminated pregnancies, this time using RNA-*in situ* hybridization and scanning electron microscopy. A low but consistent 16S rRNA gene signal was detected through qPCR, and DNA sequencing, and *Staphylococcus*, *Lactobacillus*, and *Gardnerella*, among other bacteria, were cultured from fetal intestinal tissues. However, some of the results of these studies have been challenged. For example, there are concerns that the 16S rRNA gene sequence data reported in the first study (87) were influenced by batch effects, and that the micrographs of bacteria-like structures did not actually reveal bacterial cells (93) [but see reply by (129)]. A more general concern with both studies is that microorganisms may have been introduced to the sampled fetuses during the delivery process. This is why the *in utero* colonization hypothesis can only be evaluated using samples obtained from cesarean deliveries without labor (69, 86, 130). For example, in a recent study of human fetal meconium obtained from rectal swabs during non-labored elective cesarean deliveries (130), the 16S rRNA gene profiles of meconium samples and technical controls were indistinguishable. Cultures of fetal meconium yielded isolates of only *Staphylococcus* and *Propionibacterium* (*Cutibacterium*), typical commensal skin bacteria (122), and there was generally no matching 16S rRNA gene signal for these bacteria in the meconium samples, suggesting they were contaminants. Therefore, the *in utero* colonization hypothesis remains in question.

Nevertheless, if there is *in utero* colonization and a viable fetal microbiota, there are two potential sources: hematogenous transfer through the placenta and ingestion of colonized amniotic fluid (75, 86, 131). Recent studies reported that both the human placenta (70–78, 80–82) and amniotic fluid (75, 83–85) consistently harbor resident bacterial communities. However, the vast majority of these studies have relied principally on data obtained from DNA sequencing technologies, and when samples possess either low or no microbial biomass, they are susceptible to the influence of background DNA contamination (103, 132–134). Indeed, other recent studies that have emphasized the identification of likely DNA contamination have concluded that there is not consistent evidence of resident bacterial communities in the human placenta (133, 135–141) or amniotic fluid (104, 105).

While the *in utero* colonization hypothesis and the existence of a placental and/or amniotic fluid microbiota have been frequently investigated in humans, they have been only rarely investigated in mice (86, 101, 107, 142). An initial study revealed bacterial signals in the placenta and fetal intestine of mice through 16S rRNA gene qPCR, and sequencing, and suggested the placenta as the likely origin of fetal bacterial DNA (101). In a second study (86), bacteria were visualized in the fetal intestine of mice through fluorescence *in situ* hybridization. Bacteria were also detected in fetal tissues through 16S rRNA gene sequencing, and potential sources of these signals were suggested to be the placenta and amniotic membranes. Furthermore, culture of fetal tissues yielded bacterial isolates, primarily *Lactobacillus* (86). However, subsequent studies have yielded no consistent evidence of a placental or a fetal microbiota in mice (107, 142). For instance, we attempted to characterize the placental and fetal (brain, lung, liver, intestine) microbiotas of mice using 16S rRNA gene qPCR, sequencing, and culture (107). Bacterial loads of placental and fetal tissues did not exceed those of technical controls, nor did they yield substantive 16S rRNA gene sequence libraries. Recovery of bacteria from placental and fetal tissues through culture was rare. For example, culture of the fetal intestine yielded only a single isolate of *Staphylococcus hominis*, a common human commensal skin bacterium (122) thus, a likely contaminant. Therefore, as is the case with human investigations, investigations of the *in utero* colonization hypothesis and of placental and fetal microbiotas in mice have yielded disparate results.

In the current study, we focused specifically on whether there is a viable microbiota in murine amniotic fluid, which has not been previously evaluated. Quantitative PCR showed a significantly greater 16S rRNA gene signal in both proximal and distal amniotic fluid samples than in the negative controls, indicating the presence of 16S rRNA gene copies in amniotic fluid samples regardless of proximity to the cervix. These findings are consistent with the qPCR results of a prior study of cattle amniotic fluid (124).

Our investigation using 16S rRNA gene sequencing detected higher relative abundances of DNA from *Corynebacterium* spp., *Pseudomonas*, *Sphingobium*, and *Streptococcus* in the amniotic fluid of mice than in technical controls (**Figure 3**). *Corynebacterium* spp. and *Streptococcus* spp. are resident microbiota of mammals, including humans and mice (122, 143–145). However, these microorganisms have also been identified as common bacterial DNA contaminants in studies with low microbial biomass (98, 103, 132). *Corynebacterium* spp. are aerobic, non-spore-forming, Gram-positive bacteria (119) that have been identified as members of the mouse skin (143) and respiratory (144) microbiotas. Specifically, *Corynebacterium tuberculostearicum* (ASV 10) has been previously detected in human amniotic fluid using molecular techniques; however, this bacterium was not recovered using conventional culture methods (84, 146). The *Streptococcus* ASV detected in the current study (ASV 13) had an identical sequence match with multiple members of the Mitis group of the genus *Streptococcus*, which are common inhabitants of the oral cavity and upper respiratory tract in humans (147) and have been detected in the lungs of mice (107). *Pseudomonas* is widely distributed amongst mammals and the broader environment (148). In our study, BLAST analysis was performed on ASV 24 (*Pseudomonas*), but a species-level taxonomy could not be assigned, indicating that the V4 region of the 16S rRNA gene is not adequate for differentiation of the *Pseudomonas* species, at least those detected herein. *Sphingobium* is typically an environmental microorganism (149). In the current study, BLAST analysis for ASV 33 showed that it was identical to the typical soil bacteria *S. naphthae*, *S. olei*, and *S. soli* (150–152). A single case was reported of *S. olei* causing peritonitis *via* infection of an indwelling peritoneal catheter in a patient with end stage renal disease (153). In summary, although some of these microorganisms have been found in biologically relevant sites, the importance of their DNA signal in amniotic fluid in this study requires further investigation.

An inherent limitation of molecular investigations is the inability to differentiate between whether the presence of 16S rRNA gene signals are due to the presence of viable bacterial communities, dead or metabolically inactive bacterial cells, bacterial cells engulfed or entrapped by host immune cells (154), and/or environmental DNA (103). While many studies have used molecular techniques to confirm the existence of bacterial DNA in the placenta, fetal tissue, and amniotic fluid (48, 75, 83–85, 96–98, 104, 105, 121), only some have attempted to culture bacteria from these same samples (75, 96, 105, 121). Notably, *Corynebacterium*, *Pseudomonas*, *Sphingobium*, *Streptococcus*, and other prominent bacteria identified in molecular surveys were not recovered in culture in this study. Indeed, the only microorganism that was cultured, *Lactobacillus murinus*, was cultured from only one mouse and it was not detected in the 16S rRNA gene profile of any amniotic fluid sample in the study. *L*. *murinus* is known to reside in the GI system of mice, where it has been documented to play a role in attenuating inflammation (155). Indeed, in a prior study (107), *L. murinus* was found in multiple body sites of pregnant mice. Given its wide distribution among and within mice, this *Lactobacillus* isolate may represent a culture cross-contaminant from other murine body sites.

### Strengths of This Study

The current study has three principal strengths. First, we used multiple, complementary modes of inquiry, including 16S rRNA gene qPCR, 16S rRNA gene sequencing, and bacterial culture to assess whether there is an amniotic fluid microbiota in mice. Furthermore, the culture component of the study included molecular validation. Second, we utilized robust sterile techniques as well as negative, experimental, and positive controls when performing extractions and molecular work to assure that any bacterial DNA signal detected in the experimental samples could be correctly attributed to a true 16S rRNA gene signal in the amniotic fluid versus environmental or reagent contamination. Third, we sampled amniotic fluid from amniotic sacs proximal and distal to the cervix to assess differential presence of microorganisms throughout the uterine horns of mice.

### Limitations of This Study

The current study has two principal limitations. First, this study focused exclusively on assessing the presence of bacteria in murine amniotic fluid; viruses and eukaryotic microorganisms were not considered in this study. Second, we used the mouse as a model to evaluate the existence of an amniotic fluid microbiota. Given that there are differences in the physiology and morphology of murine and human reproductive tracts and intra-amniotic environments (156–158), extrapolation of the findings of this study directly to humans may not be appropriate. Also, this study was conducted using a single mouse strain. Future research should consider the potential effect of variation in physiological and morphological characteristics among mouse strains and across animal facilities. Nevertheless, it is important to note that the evaluation of an amniotic fluid microbiota and *in utero* colonization does not only have translational relevance for human medicine but is also critical for our understanding of mammalian developmental and evolutionary biology in general (69, 159).

## Conclusion

Using qPCR, 16S rRNA gene sequencing, and bacterial culture, we did not find consistent or reproducible evidence of an amniotic fluid microbiota in mice. This study provides evidence against amniotic fluid as a source of microorganisms for colonization of the fetus and illustrates the importance of implementing multiple methodologies and the appropriate technical controls in investigations assessing microbial profiles of body sites historically presumed to be sterile. However, although this study indicates that the DNA signals detected in murine amniotic fluid samples were not derived from a viable microbiota, this finding does not preclude the importance of *in utero* exposure to the components and/or products of microorganisms for mammalian fetal development (100, 160). This potentiality warrants further investigation.

## Data Availability Statement

The datasets presented in this study can be found in online repositories. The names of the repository/repositories and accession number(s) can be found below: https://www.ncbi.nlm.nih.gov/, BioProject ID PRJNA751620.

## Ethics Statement

The animal study was reviewed and approved by Institutional Animal Care and Use Committee (IACUC) (Protocol No. 18-03-0584).

## Author Contributions

KT, NG-L, and RR co-conceived the study. AW, JMG, JG, VG-F, and DK performed the laboratory work and data processing. AW, ZS, and KT completed statistical analysis. AW, ZS, JG, NG-L, and KT co-wrote the manuscript. All authors read, edited, and approved the manuscript.

## Funding

This research was supported, in part, by the Perinatology Research Branch (PRB), Division of Intramural Research, *Eunice Kennedy Shriver* National Institute of Child Health and Human Development, National Institutes of Health, U. S. Department of Health and Human Services (NICHD/NIH/DHHS), and, in part, with federal funds from the NICHD/NIH/DHHS under Contract No. HHSN275201300006C. This research was also supported by the Wayne State University Perinatal Initiative in Maternal, Perinatal and Child Health. The funders had no role in the study design, data collection and analysis, decision to publish, or preparation of the manuscript. Dr. Romero has contributed to this work as part of his official duties as an employee of the United States Federal Government.

## Conflict of Interest

The authors declare that the research was conducted in the absence of any commercial or financial relationships that could be construed as a potential conflict of interest.

## Publisher’s Note

All claims expressed in this article are solely those of the authors and do not necessarily represent those of their affiliated organizations, or those of the publisher, the editors and the reviewers. Any product that may be evaluated in this article, or claim that may be made by its manufacturer, is not guaranteed or endorsed by the publisher.
