# Supplementary material for: Does the Amniotic Fluid of Mice Contain a Viable Microbiota?
Source: Front Immunol. 2022 Feb 28;13:820366. doi: 10.3389/fimmu.2022.820366 (PMC8920496; doi:10.3389/fimmu.2022.820366)
Supplement: Supplementary file 1 [file DataSheet_1.pdf]

**Does the amniotic fluid of mice contain a viable microbiota?**

Andrew D. Winters[1-3], Roberto Romero[1,4-7], Jonathan M. Greenberg[1,8], Jose Galaz[1,8], Zachary Shaffer[1,9,10], Valeria Garcia-Flores[1,8], David J. Kracht[1,8], Nardhy Gomez-Lopez[1-3,8*,†], and Kevin R. Theis[1-3,8*,†]

## Author affiliations

[1] Perinatology Research Branch, Division of Obstetrics and Maternal-Fetal Medicine, Division of Intramural Research, *Eunice Kennedy Shriver* National Institute of Child Health and Human Development, National Institutes of Health, U.S. Department of Health and Human Services, Bethesda, MD, and Detroit, MI, United States
[2] Perinatal Research Initiative in Maternal, Perinatal and Child Health, Wayne State University School of Medicine, Detroit, MI, United States
[3] Department of Biochemistry, Microbiology, and Immunology, Wayne State University School of Medicine, Detroit, MI, United States
[4] Department of Obstetrics and Gynecology, University of Michigan, Ann Arbor, MI, United States
[5] Department of Epidemiology and Biostatistics, Michigan State University, East Lansing, MI, United States
[6] Center for Molecular Medicine and Genetics, Wayne State University, Detroit, MI, United States
[7] Detroit Medical Center, Detroit, MI, United States
[8] Department of Obstetrics and Gynecology, Wayne State University School of Medicine, Detroit, MI, United States
[9] Department of Physiology, Wayne State University School of Medicine, Detroit, MI, United States
[10] MD/PhD Combined Degree Program, Wayne State University School of Medicine, Detroit, MI, United States
[†] These authors have contributed equally to this work and share senior authorship

## Corresponding author(s)

[*,†] Kevin R. Theis, ktheis@med.wayne.edu

[*,†] Nardhy Gomez-Lopez, ngomezlo@med.wayne.edu

**SUPPLEMENTARY FIGURE 1. Amniotic fluid 16S rRNA gene sequencing results before and after the removal of likely contaminating sequences in proximal and distal amniotic fluid samples.** Bars indicate the taxonomic classifications of the 20 amplicon sequence variants (ASVs) with highest relative abundance across all proximal and distal amniotic samples for each group (before and after removal of likely contaminants). Bars of identical color within the same sample indicate multiple ASVs with the same bacterial taxonomic classification.

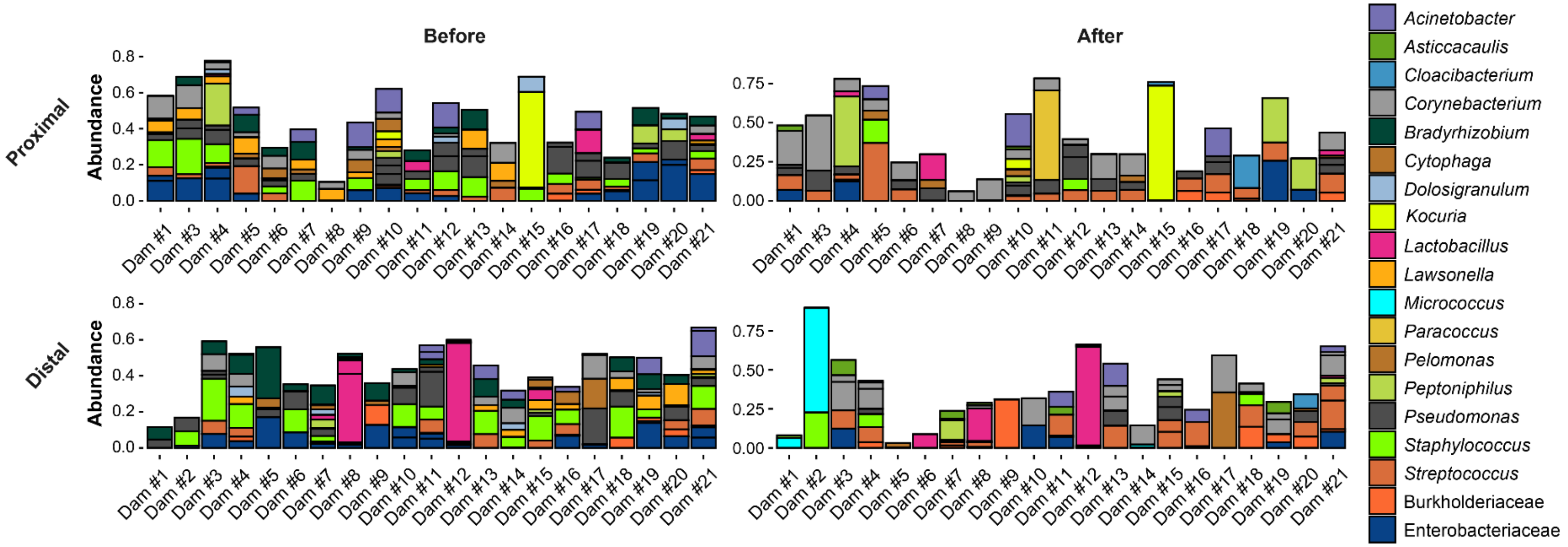

## SUPPLEMENTARY TABLES

### Supplementary Table 1. Description of prior molecular investigations of a potential microbiota in human amniotic fluid.

| Authors & year | Sample size & collection | Culture conditions | qPCR target | Sequencing target & prominent bacteria | Controls for DNA contamination | Concluded existence of an amniotic fluid microbiota |
|---|---|---|---|---|---|---|
| Rodriguez et al. 2011[1] | N=121 amniotic fluid from amniocentesis during gestational weeks 16-20 | Culture medium specific for *Ureaplasma* spp.<br><br>No cultivable biomass from amniotic fluid | N/A | No sequencing done<br><br>Multiplex endpoint polymerase chain reaction of *Ureaplasma* specific urease gene for identification *of U. parvum* and *U. urealyticum* | N/A | YES |
| Rautava et al. 2012 [2] | N=14<br>Uncomplicated pregnancies at term<br><br>Amniotic fluid collected during elective caesarean delivery | N/A | *Lactobacillus*, *Bifidobacterium*, *Bacteroides*, *Clostridium leptum* and *Clostridium coccoides* | N/A | N/A | YES |
| Collado et al. 2016 [3] | N=15<br>Healthy full-term women<br><br>Amniotic fluid collected during elective caesarean delivery | Gifu anaerobic and LB media under anoxic atmospheres<br><br>Isolated:<br>*Staphylococcus*<br>*Propionibacterium*<br>Lachnospiraceae<br>*Streptomyces* | N/A | 16S rRNA gene<br><br>Enterobacteriaceae,<br>*Enterobacter*<br>*Escherichia*<br>*Propionibacterium*<br>*Lactobacillus*<br>*Streptococcus*<br>*Staphylococcus* | N/A | YES |
| Lim et al. 2018 [4] | N=24 Uncomplicated pregnancies at term<br><br>AF collected during elective caesarean | N/A | 16S rRNA gene | 16S rRNA gene | Blank DNA extraction kits (N=4) were sequenced. | NO |

| | | | | | | |
|---|---|---|---|---|---|---|
| Rehbinder et al.<br>2018 [5] | N=10 Uncomplicated pregnancies at term<br><br>Amniotic fluid collected during elective caesarean delivery | BHI medium under oxic and anoxic atmospheres<br><br>No cultivable biomass from healthy amniotic fluid | 16S rRNA gene | 16S rRNA gene | Two negative controls were sequenced. | NO |
| Zhu et al.<br>2018 [6] | N=64 amniotic fluid samples<br><br>17-24 weeks karyotype amniocentesis<br><br>N=50 for culture | BHI and Columbia Blood media under oxic atmosphere<br><br>No cultivable biomass from amniotic fluid | N/A | 16S rRNA gene<br><br>*Propionibacterium,*<br>Bacillales<br>*Anoxybacillus*<br>Caulobacteraceae<br>Methylobacteriaceae<br>*Methylobacterium*<br>*Phyllobacterium*<br>*Sphingomonas*<br>Comamonadaceae<br>*Deinococcus*<br>Corynebacteriaceae<br>Streptococcaceae | N/A | YES |

| | | | | | | |
|---|---|---|---|---|---|---|
| Stinson et al.<br>2019 [7] | N=43<br>Uncomplicated pregnancies at 34-42 weeks gestation<br><br>Amniotic fluid collected during elective caesarean delivery | N/A | N/A | 16S rRNA gene<br><br>*Propionibacterium*<br>*Staphylococcus*<br>*Ralstonia*<br>*Streptococcus*<br>*Peptoniphilus*<br>*Corynebacterium* spp. | Blank DNA extraction kits (N=5) were sequenced. | YES |
| Stinson et al.<br>2020 [8] | N=18<br>Amniocentesis at 14-20 gestational weeks | N/A | 16S rRNA gene<br><br>Secondarily,<br>*Ureaplasma* | 16S rRNA gene<br><br>Saccharibacteria<br>*Acidovorax temperans*<br>*Tepidimonas taiwanensis*<br>*Pelomonas puraquae*<br>*Corynebacterium*<br>*Streptococcus*<br>*Pseudomonas* | Blank DNA extraction kits (N=8) were sequenced. | YES |
| Campisciano et al.<br>2021 [9] | N=29<br>Amniocentesis at 15-21 weeks | N/A | N/A | 16S rRNA gene<br><br>*Acinetobacter*<br>*Bacillus*<br>*Stenotrophomonas*<br>*Gemella*<br>*Lactobacillus*<br>*Mycoplasma*<br>*Neisseria*<br>*Ureaplasma*<br>*Veillonella* | Blank DNA extraction kits (N=7) and a sterile swab were sequenced. | YES |
| Wu et al.<br>2021 [10] | N=25, Healthy, full-term women<br><br>Amniotic fluid collected during elective caesarean delivery | N/A | N/A | 16S rRNA gene<br><br>*Sphingomonas*<br>*Staphylococcus*<br>*Streptococcus* | N/A | YES |

**Supplementary Table 2. Description of prior molecular investigations of a potential microbiota in the amniotic fluid of animal models.**

| Animal model | Authors & year | Sample size & collection | Culture conditions | qPCR target | Sequencing target & prominent bacteria | Controls for DNA contamination | Concluded existence of an amniotic fluid microbiota |
|---|---|---|---|---|---|---|---|
| Rat | Borghi et al. 2019 [11] | N = 5 pups from 2 dams<br><br>Gestational day 16/23<br><br>Cesarean delivery | N/A | N/A | 16S rRNA gene<br><br>Lachnospiraceae<br>Ruminococcaceae<br>Bacteroidaceae<br>Veillonellaceae<br>Rikenellaceae | Some blank extraction controls were sequenced and "known environmental contaminants were never observed." | YES |
| Cattle | Moore et al. 2017 [12] | N = 5 calves<br><br>Third trimester<br><br>Obtained following slaughter of cows | N/A | N/A | 16S rRNA gene<br><br>Clostridiales<br>Ruminococcaceae<br>S24-7<br>Lachnospiraceae<br>*Flavobacterium* | N/A | YES |
| Cattle | Guzman et al. 2020 [13] | N = 12 calves<br><br>5, 6, or 7 months / 9.4 months<br><br>Obtained following slaughter of cows | N/A | 16S rRNA gene | 16S rRNA gene<br><br>Flavobacteriales<br>Rhodobacterales<br>Xanthomonadales<br>Enterobacteriales<br>Sphingomonadales<br>Pseudomonadales | Two blank extraction controls were sequenced, and these data were compared to amniotic fluid profiles (there was minimal overlap). | YES |
| Cattle | Husso et al. 2021 [14] | N = 23 calves<br><br>Term gestation<br><br>Cesarean delivery prior to any rupture of membranes | Gifu Anaerobic Medium Agar under oxic and anoxic atmospheres | 16S rRNA gene | 16S rRNA gene<br><br>*Staphylococcus**<br>*Streptococcus*<br>*Delftia*<br>*Sphingomonas**<br>*Enterococcus*<br><br>**Staphylococcus* and *Sphingomonas* were more relatively abundant in the profiles of amniotic fluid than the meconium. | Eight nuclease-free water controls were sequenced. *Decontam* was run. | NO |
| Sheep | Malmuthuge & Griebel 2018 [15] | N = 16 lambs<br><br>Gestation day 125-135/144-152 | N/A | 16S rRNA gene | 16S rRNA gene | One PCR control was sequenced. | NO |

| | | | | | | | |
|---|---|---|---|---|---|---|---|
| | | Cesarean delivery | | | | | |
| Goat | Zou et al. 2020 [16] | N = 3<br><br>Gestational day 90, 100, or 120 / 145-152<br><br>Cesarean delivery | N/A | N/A | 16S rRNA gene<br><br>Comamonadaceae<br>Burkholderiales | Three blank extraction controls were sequenced and these data were used to determine which sequences should be removed from the dataset. | YES |
| Horse | Quercia et al. 2019 [17] | N = 13 foals<br><br>Gestational day 333-355/330-360<br><br>Vaginal delivery (needle puncture of the exposed amnion) | N/A | N/A | 16S rRNA gene<br><br>*Pseudomonas*<br>*Sphingomonas*<br>*Enterococcus*<br>*Staphylococcus*<br>*Erwinia*<br>*Pedobacter* | None. However, previously identified contaminants constituted only a fraction of the bacterial profiles of amniotic fluid. | YES |

## REFERENCES


1. Rodríguez, N., et al., *Detection of Ureaplasma urealyticum and Ureaplasma parvum in amniotic fluid: association with pregnancy outcomes.* J Matern Fetal Neonatal Med, 2011. **24**(1): p. 47-50.
2. Rautava, S., et al., *Probiotics modulate host-microbe interaction in the placenta and fetal gut: a randomized, double-blind, placebo-controlled trial.* Neonatology, 2012. **102**(3): p. 178-84.
3. Collado, M.C., et al., *Human gut colonisation may be initiated in utero by distinct microbial communities in the placenta and amniotic fluid.* Sci Rep, 2016. **6**: p. 23129.
4. Lim, E.S., C. Rodriguez, and L.R. Holtz, *Amniotic fluid from healthy term pregnancies does not harbor a detectable microbial community.* Microbiome, 2018. **6**(1): p. 87.
5. Rehbinder, E.M., et al., *Is amniotic fluid of women with uncomplicated term pregnancies free of bacteria?* Am J Obstet Gynecol, 2018. **219**(3): p. 289.e1-289.e12.
6. Zhu, L., et al., *Bacterial Communities in the Womb During Healthy Pregnancy.* Front Microbiol, 2018. **9**: p. 2163.
7. Stinson, L.F., et al., *The Not-so-Sterile Womb: Evidence That the Human Fetus Is Exposed to Bacteria Prior to Birth.* Front Microbiol, 2019. **10**: p. 1124.
8. Stinson, L., et al., *Comparison of Bacterial DNA Profiles in Mid-Trimester Amniotic Fluid Samples From Preterm and Term Deliveries.* Front Microbiol, 2020. **11**: p. 415.
9. Campisciano, G., et al., *Evidence of bacterial DNA presence in chorionic villi and amniotic fluid in the first and second trimester of pregnancy.* Future Microbiol, 2021. **16**: p. 801-810.
10. Wu, S., et al., *Do Maternal Microbes Shape Newborn Oral Microbes?* Indian J Microbiol, 2021. **61**(1): p. 16-23.
11. Borghi, E., et al., *Antenatal Microbial Colonization of Mammalian Gut.* Reprod Sci, 2019. **26**(8): p. 1045-1053.
12. Moore, S.G., et al., *Hot topic: 16S rRNA gene sequencing reveals the microbiome of the virgin and pregnant bovine uterus.* J Dairy Sci, 2017. **100**(6): p. 4953-4960.
13. Guzman, C.E., et al., *A pioneer calf foetus microbiome.* Sci Rep, 2020. **10**(1): p. 17712.
14. Husso, A., et al., *The Composition of the Microbiota in the Full-Term Fetal Gut and Amniotic Fluid: A Bovine Cesarean Section Study.* Front Microbiol, 2021. **12**: p. 626421.
15. Malmuthuge, N. and P.J. Griebel, *Fetal environment and fetal intestine are sterile during the third trimester of pregnancy.* Vet Immunol Immunopathol, 2018. **204**: p. 59-64.
16. Zou, X., et al., *Exploring the Rumen and Cecum Microbial Community from Fetus to Adulthood in Goat.* Animals (Basel), 2020. **10**(9).
17. Quercia, S., et al., *Early colonisation and temporal dynamics of the gut microbial ecosystem in Standardbred foals.* Equine Vet J, 2019. **51**(2): p. 231-237.
